# Factors Associated With First Occurrences of Child Maltreatment in Military Families

**DOI:** 10.1001/jamanetworkopen.2026.12199

**Published:** 2026-05-13

**Authors:** Stephen J. Cozza, Christin M. Ogle, Joscelyn E. Fisher, Jing Zhou, Sierra L. Martin, James A. Naifeh, Holly B. H. Mash, Pablo A. Aliaga, Carol S. Fullerton, Robert J. Ursano

**Affiliations:** 1 Center for the Study of Traumatic Stress, Department of Psychiatry, Uniformed Services University of the Health Sciences, Bethesda, Maryland; 2 Henry M. Jackson Foundation for the Advancement of Military Medicine, Inc, Bethesda, Maryland

## Abstract

**Importance:**

Information regarding risk factors among service member families for different child maltreatment types is needed to improve prevention efforts.

**Objective:**

To identify factors associated with first occurrences of 4 child maltreatment types and examine child age–related changes in risk for each type among active duty service member families.

**Design, Setting, and Participants:**

This population-based retrospective cohort study used data from the Child Maltreatment in Military Families Life Course Study on active duty service member families with a first occurrence of child maltreatment in fiscal years 2009 through 2018 and a representative sample of active duty families without child maltreatment incidents. Data were analyzed from August 2023 to February 2026.

**Exposure:**

One or more parents serving as an active duty service member.

**Main Outcomes and Measures:**

First documented occurrences of neglect, physical abuse, emotional abuse, and sexual abuse, identified using Family Advocacy Program data. Associations between sociodemographic, family, and military-related characteristics and first occurrences of maltreatment types were examined in univariable and multivariable logistic regressions.

**Results:**

The study included 618 101 active duty service member families (28 684 [4.64%] with a first occurrence of child maltreatment and 589 417 [95.36%] without child maltreatment incidents), consisting of 1 070 510 family-months (FM); the total weighted sample was 65 142 809 FM (59 031 293 male service member FM [90.62%]; mean [SD] age, 32.74 [6.97] years). Crude rates of child maltreatment were highest for child neglect (22.16 per 100 000 FM) followed by physical abuse (10.97 per 100 000 FM), emotional abuse (4.23 per 100 000 FM), and sexual abuse (2.66 per 100 000 FM). Factors associated with higher odds of all child maltreatment types in multivariable models were female service member families (eg, sexual abuse: odds ratio [OR], 1.39 [95% CI, 1.18-1.64]; physical abuse: OR, 1.82 [95% CI, 1.70-1.95]), early parenting (age <21 years) (eg, neglect: OR, 1.32 [95% CI, 1.22-1.41]; sexual abuse: OR, 2.12 [95% CI, 1.75-2.56]), larger number of dependent children (≥3) (eg, emotional abuse: OR, 1.63 [95% CI, 1.48-1.79]; sexual abuse: OR, 2.32 [95% CI, 2.06-2.61]), and never-deployed status (eg, sexual abuse: OR, 1.90 [95% CI, 1.55-2.32]; emotional abuse: OR, 3.76 [95% CI, 3.09-4.57]). Risk rates peaked at 3 months of age for neglect (48.61 per 100 000 FM) and physical abuse (25.49 per 100 000 FM). Risk of emotional and sexual abuse peaked in middle childhood (age 5-12 years) and adolescence but was generally lower (<6 per 100 000 FM) than risk of other child maltreatment types across all ages.

**Conclusions and Relevance:**

In this cohort study, families with female service members, never-deployed service members, 3 or more children, and young parents had higher risk of child maltreatment. Dynamic prevention approaches appear to be needed to address evolving risk factors across the family life course.

## Key Points

**Question:**

What factors are associated with first occurrences of 4 child maltreatment types (neglect and physical, emotional, and sexual abuse) in service member families?

**Findings:**

In this cohort study of 618 101 active duty service member families, families with a female service member, a young service member at the transition to parenthood, 3 or more children, and never-deployed status had greater risk of all maltreatment types.

**Meaning:**

These findings suggest that tailored prevention efforts are needed to address risk conditions in families with female service members, young service member parents, a larger family size, and never-deployed service members.

## Teaser

This cohort study examines factors associated with first occurrences of child neglect and physical, emotional, and sexual abuse among active duty service member families.

## Introduction

Child maltreatment is associated with multiple negative mental and physical health outcomes, including psychiatric disorders,^1,2^ suicide,^3^ and chronic health conditions.^4,5^ Identifying risk factors for child maltreatment is critical to advancing targeted prevention efforts that reduce children’s risk of harm. However, prior research has primarily examined factors associated with single types of abuse or neglect in civilian families.^6,7,8^ Findings from civilian samples may not generalize to military families who are exposed to unique stressors (eg, frequent relocations, deployments) and protective factors (eg, health care, housing allowance, and subsidized childcare) that may differentially impact child maltreatment types. An investigation of risk factors for different child maltreatment types in military families is needed to advance understanding of the conditions associated with elevated risk of particular child maltreatment types to inform prevention strategies tailored to specific high-risk groups.

Understanding the ages at which children are at higher risk for specific types of child maltreatment is also critical to effective prevention. Existing research has been primarily limited to temporal changes in overall child maltreatment^9,10^ or single maltreatment types.^11^ For example, national data on the age of children involved in substantiated incidents indicate that risk of any type is greatest during the first year of life.^12^ Less is known about differences in risk for distinct maltreatment types in military families. A more fine-grained examination of age-related changes in risk is needed to identify when children are at heightened risk for particular child maltreatment types and to inform targeted prevention efforts that address the needs of families during specific high-risk periods of the military family life course.

The present study used population-level administrative data on active duty military service member families to identify factors associated with first occurrences of 4 child maltreatment types (neglect, physical abuse, emotional abuse, and sexual abuse). Consistent with ecological models of child maltreatment,^13,14^ factors were selected from multiple levels of the child’s social ecology (parent, family, and military) based on empirical evidence of their association with increased risk of child maltreatment in civilian^6,7,8^ and military families.^15,16^ Although covariation among the selected factors was expected, each was hypothesized to be associated with distinct risk for child maltreatment. For instance, although younger parental age is likely correlated with lower military rank, parental age was included to index the unique risks associated with early parenting; rank was included as an indicator of socioeconomic status and differences in occupational exposures across ranks that may be differentially associated with child maltreatment types. Child age-related changes in risk of child maltreatment types were also examined. Results are expected to advance understanding of differences in factors associated with child maltreatment types, the characteristics of families with high risk of child maltreatment that should be prioritized for preventive services, and when children are at higher risk for each maltreatment type across the military family life course.

## Methods

### Sample

Data for this cohort study were drawn from the Child Maltreatment in Military Families Life Course Study, a longitudinal, retrospective cohort study that integrates person-month records for service member sponsors and their dependents.^17^ A sponsor is the service member parent whose military status determines the eligibility of their dependents’ military benefits (eg, health care). Records for all regular component active duty enlisted and officer service member sponsors with 1 or more children in fiscal years (FYs) 2009 to 2018 were merged with records for their spouse and child dependents to create family-month (FM) records. Each FM represented a given family at a specific month in FYs 2009 to 2018. Families with a documented child maltreatment incident that met US Department of War (DOW) criteria for child abuse or neglect^17^ prior to FY 2009 were excluded. A case FM represented the month of the child maltreatment incident report date for the families with a first documented occurrence of child maltreatment that met DOW criteria for child maltreatment^17^ in FYs 2009 to 2018. To reduce computational intensity, FM for families without a documented child maltreatment incident during FYs 2009 to 2018 were selected using an equal probability stratified sampling procedure from the population of active duty families with 1 or more children younger than 18 years in FYs 2009 to 2018 and were defined as at-risk FM. Additional details regarding the sampling procedure and the discrete-time analytic dataset are in Ogle et al.^17^ The study was approved by the institutional review board at the Uniformed Services University of the Health Sciences. Informed consent was waived because the study used deidentified data. The study followed the Strengthening the Reporting of Observational Studies in Epidemiology (STROBE) reporting guideline.

### Measures

Data about child maltreatment incidents were drawn from the Family Advocacy Program Central Registry, a DOW database that contains information on child abuse and neglect incidents involving service members or their dependents. Data on examined sociodemographic, family, and military-related characteristics were obtained from the Active Duty Family, the Active Duty Military Personnel Master, the Defense Enrollment Eligibility Reporting System, and the Contingency Tracking System-Overseas Contingency Operations data files.

#### Outcome

First occurrence of child maltreatment was defined as the first documented child maltreatment incident in a family (inclusive of all child survivors of maltreatment, child maltreatment types, and perpetrators of maltreatment) that occurred in FYs 2009 to 2018 and met the DOW criteria for child abuse (emotional, physical, and sexual) or neglect.^18^ The eAppendix in Supplement 1 includes complete DOW criteria. Analyses included incidents that exclusively involved neglect, emotional abuse, physical abuse, or sexual abuse.

#### Sociodemographic, Family, and Military-Related Characteristics

Sociodemographic characteristics of the sponsor (or higher-ranking service member in dual-military couples) included sex, age at birth of oldest child, and current age. Family characteristics included marital status, children at entry into active duty (yes or no), age of youngest child, and number of dependent children. Military-related characteristics included military rank of the sponsor (or the higher-ranking service member in dual-military couples), which served as a proxy measure of household income, and deployment status (never deployed, ever deployed, and currently deployed), which indexed the combat deployment history of the sponsor (or both parents in dual-military couples) in relation to the case FM or at-risk FM.

### Statistical Analysis

Analyses used a discrete time survival analysis framework, with FM examined as the unit of analysis. At-risk FM were assigned a weight of 62.5, the inverse probability of selection (Ogle et al^17^). Associations between sociodemographic, family, and military-related factors and first occurrences of maltreatment types were examined in univariable and multivariable logistic regressions adjusted for all factors, time since first at-risk month, fiscal year, calendar month, and select covariates that were expected to be associated with child maltreatment risk, including sponsor race (American Indian or Alaska Native, Asian, Black, Native Hawaiian or Other Pacific Islander, White, and other [multiracial or unknown]), ethnicity (Hispanic and non-Hispanic), educational level (less than high school, high school or equivalent, some college, and college degree or higher), and military branch (Air Force, Army, Marine Corps, and Navy). Race and ethnicity were drawn from the Active Duty Military Personnel Master data file and used as covariates because they were expected to be associated with child maltreatment risk. Separate models were conducted for distinct maltreatment types. For categorical factors, the largest stratum was selected as the reference group except for number of dependent children, deployment status, and service branch. Wald χ^2^ tests, odds ratios (ORs), and 95% CIs were examined to identify statistically significant factors. Mean estimates of all categories of each factor were used to generate standardized rate estimates,^19^ which represent rates of first occurrences of child maltreatment per 100 000 FMs assuming other factors were at the samplewide mean.

To examine risk of first occurrences of each maltreatment type as a function of time since first at-risk month, linear spline analyses^20^ were conducted, with knots representing changes in slope identified using an empirically derived, iterative process. This analysis included the subset of families in which the first biological child was born on or after the service member entered active duty. Fit statistics, including Akaike information criterion, Schwarz criterion, and log likelihood, were used to assess model fit and knot placement. Monthly risk rates calculated from discrete time models were plotted to illustrate changes in risk of first occurrences of each child maltreatment type in families following the birth of the first biological child.

Statistical significance was set at *P* < .05 (2-tailed). Data were analyzed from August 2023 to February 2026. All statistical analyses were performed using SAS, version 9.4 (SAS Institute Inc).^21^

## Results

The study included 618 101 active duty service member families, representing 1 070 510 FM. The total weighted sample was 65 142 809 FM, with 6 110 954 female (9.38%) and 59 031 293 male (90.62%) service member FM. Mean (SD) age of service members was 32.74 (6.97) years. A total of 28 684 service member families (4.64%) had a first occurrence of child maltreatment, and 589 417 (95.36%) did not have a child maltreatment incident in our sampled data. A total of 885 075 FM (1.37%) were American Indian or Alaska Native, 2 386 252 FM (3.70%) were Asian, 12 171 484 FM (18.89%) were Black, 591 404 FM (0.92%) were Native Hawaiian or Other Pacific Islander, 43 741 887 FM (67.90%) were White, and 4 643 749 FM (7.21%) were other race; 7 989 216 FM (12.54%) were Hispanic, and 55 709 353 FM (87.46%) were non-Hispanic.

### Characteristics of First Occurrences of Child Maltreatment

The majority of first occurrences of child maltreatment involved 1 perpetrator (26 260 [91.55%]) and 1 child survivor (21 665 [75.53%]). The mean (SD) age of child survivors was 5.43 (4.63) years. Approximately half were male (19 353 [50.51%]), and 18 959 (49.49%) were female. Most perpetrators were male (17 856 [58.21%]; 12 821 [41.79%] were female) and sponsor parents (16 475 [54.32%]), with a mean (SD) age of 29.02 (6.94) years. Civilian parents constituted 31.62% of perpetrators (n = 9591). The remaining 4262 perpetrators (14.05%) included extrafamilial caregivers, nonparental family members, noncaregiver acquaintances, and unknown individuals.

Approximately half of first occurrences of child maltreatment involved neglect only (14 433 [50.32%]); 7145 (24.91%) involved physical abuse only, 2755 (9.60%) involved emotional abuse only, 1731 (6.03%) involved sexual abuse only, and 2620 (9.13%) involved multiple types. Crude rates of child maltreatment were highest for child neglect (22.16 per 100 000 FM) followed by physical abuse (10.97 per 100 000 FM), emotional abuse (4.23 per 100 000 FM), and sexual abuse (2.66 per 100 000 FM).

### Factors Associated With Child Maltreatment Types

Sponsor sociodemographic, family, and military-related characteristics are shown in Table 1. Table 2 shows results of univariable analyses of first occurrences of each maltreatment type by each examined factor. Table 3 shows results of multivariable analyses of first occurrences of each maltreatment type by each examined factor. eTables 1 to 3 in Supplement 1 give complete model results. Variance inflation factors for the covariates included in the multivariable models are shown in eTable 4 in Supplement 1.

**Table 1.** Sponsor Sociodemographic, Family, and Military-Related Characteristics by Child Maltreatment Type in Active Duty Families, Fiscal Years 2009 to 2018

Characteristic	Neglect, FM (%)		Abuse, FM (%)					
			Physical		Emotional		Sexual	
	Cases (n = 14 433)	Total (n = 65 128 558)	Cases (n = 7145)	Total (n = 65 121 270)	Cases (n = 2755)	Total (n = 65 116 880)	Cases (n = 1731)	Total (n = 65 115 856)
**Sponsor sociodemographic characteristics**								
Sex								
Female	2145 (14.86)	6 108 708 (9.38)	1273 (17.82)	6 107 836 (9.38)	395 (14.34)	6 106 958 (9.38)	190 (10.98)	6 106 753 (9.38)
Male	12 288 (85.14)	59 019 288 (90.62)	5872 (82.18)	59 012 872 (90.62)	2360 (85.66)	59 009 360 (90.62)	1541 (89.02)	59 008 541 (90.62)
Age at birth of oldest child, y^a^								
<21	5920 (41.29)	15 712 858 (24.39)	2991 (42.45)	15 709 929 (24.39)	1121 (40.97)	15 708 059 (24.39)	788 (45.76)	15 707 726 (24.39)
21-24	5671 (39.55)	21 627 296 (33.57)	2600 (36.72)	21 624 225 (33.57)	954 (34.87)	21 622 579 (33.57)	597 (34.67)	21 622 222 (33.57)
≥25	2748 (19.16)	27 084 311 (42.04)	1489 (21.03)	27 083 052 (42.04)	661 (24.16)	27 082 224 (42.04)	337 (19.57)	27 081 900 (42.04)
Current age, y^b^								
<21	638 (4.42)	773 951 (1.20)	184 (2.58)	773 497 (1.20)	25 (0.91)	773 338 (1.20)	5 (0.29)	773 318 (1.20)
21-24	4555 (31.56)	7 147 993 (11.05)	1279 (17.91)	7 144 717 (11.04)	370 (13.44)	7 143 808 (11.04)	106 (6.12)	7 143 544 (11.04)
25-29	4819 (33.39)	15 131 757 (23.38)	2002 (28.03)	15 128 940 (23.38)	742 (26.94)	15 127 680 (23.38)	397 (22.93)	15 127 335 (23.38)
30-34	2616 (18.13)	15 829 741 (24.46)	1811 (25.35)	15 828 936 (24.46)	724 (26.29)	15 827 849 (24.46)	520 (30.04)	15 827 645 (24.46)
35-39	1281 (8.88)	13 968 844 (21.58)	1216 (17.02)	13 968 779 (21.59)	559 (20.30)	13 968 122 (21.59)	462 (26.69)	13 968 025 (21.59)
≥40	522 (3.62)	11 863 897 (18.33)	651 (9.11)	11 864 026 (18.33)	334 (12.13)	11 863 709 (18.34)	241 (13.92)	11 863 616 (18.34)
**Family characteristics**								
Marital status								
Military-civilian marriage	11 888 (82.38)	53 784 451 (82.96)	5519 (77.26)	53 778 082 (82.96)	2378 (86.35)	53 774 941 (82.96)	1473 (85.10)	53 774 036 (82.96)
Dual-military marriage	729 (5.05)	3 287 354 (5.07)	589 (8.25)	3 287 214 (5.07)	134 (4.87)	3 286 759 (5.07)	88 (5.08)	3 286 713 (5.07)
Divorced, separated, or widowed	497 (3.44)	3 388 497 (5.23)	439 (6.15)	3 388 439 (5.23)	113 (4.10)	3 388 113 (5.23)	91 (5.26)	3 388 091 (5.23)
Never married	1317 (9.13)	4 370 005 (6.74)	596 (8.34)	4 369 284 (6.74)	129 (4.68)	4 368 817 (6.74)	79 (4.56)	4 368 767 (6.74)
Children at entry into active duty								
No	9075 (63.12)	47 670 013 (73.38)	4430 (62.18)	47 665 368 (73.38)	1594 (57.94)	47 662 532 (73.38)	885 (51.13)	47 661 823 (73.38)
Yes	5303 (36.88)	17 293 428 (26.62)	2694 (37.82)	17 290 819 (26.62)	1157 (42.06)	17 289 282 (26.62)	846 (48.87)	17 288 971 (26.62)
No. of dependent children								
1	5835 (40.43)	24 570 210 (37.73)	1954 (27.35)	24 566 329 (37.73)	646 (23.45)	24 565 021 (37.73)	212 (12.25)	24 564 587 (37.73)
2	4596 (31.84)	23 572 784 (36.20)	2310 (32.33)	23 570 498 (36.20)	994 (36.08)	23 569 182 (36.20)	530 (30.62)	23 568 718 (36.20)
≥3	4002 (27.73)	16 983 752 (26.08)	2881 (40.32)	16 982 631 (26.08)	1115 (40.47)	16 980 865 (26.08)	989 (57.13)	16 980 739 (26.08)
Age of youngest child, y								
0-1	7952 (55.10)	20 878 452 (32.14)	2805 (39.29)	20 873 305 (32.14)	687 (24.95)	20 871 187 (32.14)	353 (20.42)	20 870 853 (32.14)
2-4	4446 (30.80)	18 412 446 (28.35)	2108 (29.53)	18 410 108 (28.35)	926 (33.62)	18 408 926 (28.35)	544 (31.46)	18 408 544 (28.35)
5-12	1936 (13.41)	20 643 561 (31.78)	1956 (27.40)	20 643 581 (31.79)	1014 (36.82)	20 642 639 (31.79)	714 (41.30)	20 642 339 (31.79)
≥13	99 (0.69)	5 016 662 (7.72)	270 (3.78)	5 016 833 (7.72)	127 (4.61)	5 016 690 (7.73)	118 (6.82)	5 016 681 (7.73)
**Military-related characteristics**								
Military rank or pay grade								
E1-E3	2909 (20.16)	4 549 284 (6.99)	785 (10.99)	4 547 160 (6.98)	257 (9.33)	4 546 632 (6.98)	94 (5.43)	4 546 469 (6.98)
E4-E6	10 289 (71.29)	35 102 789 (53.90)	5061 (70.83)	35 097 561 (53.90)	1855 (67.33)	35 094 355 (53.89)	1213 (70.08)	35 093 713 (53.89)
E7-E9	697 (4.83)	11 422 760 (17.54)	797 (11.15)	11 422 860 (17.54)	384 (13.94)	11 422 447 (17.54)	269 (15.54)	11 422 332 (17.54)
Warrant officer	108 (0.75)	1 619 921 (2.49)	94 (1.32)	1 619 907 (2.49)	59 (2.14)	1 619 872 (2.49)	32 (1.85)	1 619 845 (2.49)
O1-O3	281 (1.95)	5 221 406 (8.02)	229 (3.21)	5 221 354 (8.02)	89 (3.23)	5 221 214 (8.02)	60 (3.47)	5 221 185 (8.02)
≥O4	149 (1.03)	7 212 149 (11.07)	179 (2.51)	7 212 179 (11.08)	111 (4.03)	7 212 111 (11.08)	63 (3.64)	7 212 063 (11.08)
Deployment status								
Currently deployed	1521 (10.54)	7 874 459 (12.09)	574 (8.03)	7 873 512 (12.09)	132 (4.79)	7 873 070 (12.09)	176 (10.17)	7 873 114 (12.09)
Ever deployed	8161 (56.54)	48 317 661 (74.18)	4898 (68.55)	48 314 398 (74.19)	2037 (73.94)	48 311 537 (74.19)	1281 (74.00)	48 310 781 (74.19)
Never deployed	4751 (32.92)	8 936 439 (13.72)	1673 (23.41)	8 933 361 (13.72)	586 (21.27)	8 932 274 (13.72)	274 (15.83)	8 931 962 (13.72)

Abbreviations: E*x*, enlisted pay grade *x*; FM, family-months; O*x*, commissioned officer pay grade *x*.

^a^ For nonbiological children, parental age at birth of oldest child was calculated based on the first month of the child’s inclusion in the Active Duty Family data file.

^b^ A time-varying variable, defined as the age of the service member sponsor at each case or at-risk month.

**Table 2.** Univariable Analysis of Sponsor Sociodemographic, Family, and Military-Related Characteristics and First Occurrence of Child Maltreatment by Type in Active Duty Families, Fiscal Years 2009 to 2018

Characteristic	Neglect		Abuse					
			Physical		Emotional		Sexual	
	Rate, per 100 000 FM	OR (95% CI)	Rate, per 100 000 FM	OR (95% CI)	Rate, per 100 000 FM	OR (95% CI)	Rate, per 100 000 FM	OR (95% CI)
**Sponsor sociodemographic characteristics**								
Sex								
Female	35.11	1.69 (1.61-1.77)^a^	20.84	2.10 (1.97-2.23)^a^	6.47	1.62 (1.45-1.80)^a^	3.11	1.19 (1.03-1.39)^b^
Male	20.82	1 [Reference]	9.95	1 [Reference]	4.00	1 [Reference]	2.61	1 [Reference]
Age at birth of oldest child, y^c^								
<21	37.68	3.71 (3.55-3.89)^a^	19.04	3.46 (3.26-3.69)^a^	7.14	2.92 (2.66-3.22)^a^	5.02	4.03 (3.55-4.58)^a^
21-24	26.22	2.58 (2.47-2.70)^a^	12.02	2.19 (2.05-2.33)^a^	4.41	1.81 (1.64-2.00)^a^	2.76	2.22 (1.94-2.54)^a^
≥25	10.15	1 [Reference]	5.50	1 [Reference]	2.44	1 [Reference]	1.24	1 [Reference]
Current age, y^d^								
<21	82.43	4.99 (4.58-5.44)^a^	23.79	2.08 (1.79-2.42)^a^	3.23	0.71 (0.47-1.05)	0.65	0.20 (0.08-0.48)^a^
21-24	63.72	3.86 (3.68-4.05)^a^	17.90	1.57 (1.46-1.68)^a^	5.18	1.13 (1.00-1.28)	1.48	0.45 (0.37-0.56)^a^
25-29	31.85	1.93 (1.84-2.02)^a^	13.23	1.16 (1.09-1.23)^a^	4.90	1.07 (0.97-1.19)	2.62	0.80 (0.70-0.91)^a^
30-34	16.53	1 [Reference]	11.44	1 [Reference]	4.57	1 [Reference]	3.29	1 [Reference]
35-39	9.17	0.56 (0.52-0.59)^a^	8.71	0.76 (0.71-0.82)^a^	4.00	0.88 (0.78-0.98)^b^	3.31	1.01 (0.89-1.14)
≥40	4.40	0.27 (0.24-0.29)^a^	5.49	0.48 (0.44-0.53)^a^	2.82	0.62 (0.54-0.70)^a^	2.03	0.62 (0.53-0.72)^a^
**Family characteristics**								
Marital status								
Military-civilian marriage	22.10	1 [Reference]	10.26	1 [Reference]	4.42	1 [Reference]	2.74	1 [Reference]
Dual-military marriage	22.18	1.00 (0.93-1.08)	17.92	1.75 (1.60-1.90)^a^	4.08	0.92 (0.78-1.10)	2.68	0.98 (0.79-1.21)
Divorced, separated, or widowed	14.67	0.66 (0.61-0.73)^a^	12.96	1.26 (1.15-1.39)^a^	3.34	0.75 (0.62-0.91)^e^	2.69	0.98 (0.79-1.21)
Never married	30.14	1.36 (1.29-1.44)^a^	13.64	1.33 (1.22-1.45)^a^	2.95	0.67 (0.56-0.80)^a^	1.81	0.66 (0.53-0.83)^a^
Children at entry into active duty								
No	19.04	1 [Reference]	9.29	1 [Reference]	3.34	1 [Reference]	1.86	1 [Reference]
Yes	30.66	1.61 (1.56-1.67)^a^	15.58	1.68 (1.60-1.76)^a^	6.69	2.00 (1.86-2.16)^a^	4.89	2.64 (2.40-2.90)^a^
No. of dependent children								
1	23.75	1.22 (1.17-1.27)^a^	7.95	0.81 (0.76-0.86)^a^	2.63	0.62 (0.57-0.69)^a^	0.86	0.38 (0.33-0.45)^a^
2	19.50	1 [Reference]	9.80	1 [Reference]	4.22	1 [Reference]	2.25	1 [Reference]
≥3	23.56	1.21 (1.16-1.26)^a^	16.96	1.73 (1.64-1.83)^a^	6.57	1.56 (1.43-1.70)^a^	5.82	2.59 (2.33-2.88)^a^
Age of youngest child, y								
0-1	38.09	19.30 (15.83-23.53)^a^	13.44	2.50 (2.20-2.83)^a^	3.29	1.30 (1.08-1.57)^e^	1.69	1 [Reference]
2-4	24.15	12.24 (10.03-14.93)^a^	11.45	2.13 (1.87-2.42)^a^	5.03	1.99 (1.65-2.39)^a^	2.96	1.75 (1.53-2.00)^a^
5-12	9.38	4.75 (3.88-5.82)^a^	9.48	1.76 (1.55-2.00)^a^	4.91	1.94 (1.61-2.33)^a^	3.46	2.05 (1.80-2.32)^a^
≥13	1.97	1 [Reference]	5.38	1 [Reference]	2.53	1 [Reference]	2.35	1.39 (1.13-1.71)^e^
**Military-related characteristics**								
Military rank or pay grade								
E1-E3	63.94	2.18 (2.09-2.27)^a^	17.26	1.20 (1.11-1.29)^a^	5.65	1.07 (0.94-1.22)	2.07	0.60 (0.49-0.74)^a^
E4-E6	29.31	1 [Reference]	14.42	1 [Reference]	5.29	1 [Reference]	3.46	1 [Reference]
E7-E9	6.10	0.21 (0.19-0.23)^a^	6.98	0.48 (0.45-0.52)^a^	3.36	0.64 (0.57-0.71)^a^	2.36	0.68 (0.60-0.78)^a^
Warrant officer	6.67	0.23 (0.19-0.28)^a^	5.80	0.40 (0.33-0.49)^a^	3.64	0.69 (0.53-0.89)^e^	1.98	0.57 (0.40-0.81)^e^
O1-O3	5.38	0.18 (0.16-0.21)^a^	4.39	0.30 (0.27-0.35)^a^	1.70	0.32 (0.26-0.40)^a^	1.15	0.33 (0.26-0.43)^a^
≥O4	2.07	0.07 (0.06-0.08)^a^	2.48	0.17 (0.15-0.20)^a^	1.54	0.29 (0.24-0.35)^a^	0.87	0.25 (0.20-0.33)^a^
Deployment status								
Currently deployed	19.32	1 [Reference]	7.29	1 [Reference]	1.68	1 [Reference]	2.24	1 [Reference]
Ever deployed	16.89	0.87 (0.83-0.92)^a^	10.14	1.39 (1.28-1.52)^a^	4.22	2.51 (2.11-3.00)^a^	2.65	1.19 (1.01-1.39)^b^
Never deployed	53.16	2.75 (2.60-2.92)^a^	18.73	2.57 (2.34-2.83)^a^	6.56	3.91 (3.24-4.72)^a^	3.07	1.37 (1.14-1.66)^e^

Abbreviations: E*x*, enlisted pay grade *x*; FM, family-months; OR, odds ratio; O*x*, commissioned officer pay grade *x*.

^a^ *P* < .001.

^b^ *P* < .05.

^c^ For nonbiological children, parental age at birth of oldest child was calculated based on the first month of the child’s inclusion in the Active Duty Family data file.

^d^ A time-varying variable, defined as the age of the service member sponsor at each case or at-risk month.

^e^ *P* < .01.

**Table 3.** Multivariable Analysis of Sponsor Sociodemographic, Family, and Military-Related Characteristics and First Occurrence of Child Maltreatment by Type in Active Duty Families, Fiscal Years 2009 to 2018^a^

Characteristic	Neglect		Abuse					
			Physical		Emotional		Sexual	
	SRE, per 100 000 FM^b^	OR (95% CI)	SRE, per 100 000 FM^b^	OR (95% CI)	SRE, per 100 000 FM^b^	OR (95% CI)	SRE, per 100 000 FM^b^	OR (95% CI)
**Sponsor sociodemographic characteristics**								
Sex								
Female	34.78	1.63 (1.55-1.71)^c^	18.58	1.82 (1.70-1.95)^c^	7.15	1.77 (1.57-1.99)^c^	3.63	1.39 (1.18-1.64)^c^
Male	21.39	1 [Reference]	10.21	1 [Reference]	4.05	1 [Reference]	2.62	1 [Reference]
Age at birth of oldest child, y^d^								
<21	24.67	1.32 (1.22-1.41)^c^	13.93	1.74 (1.58-1.92)^c^	5.09	1.44 (1.24-1.68)^c^	3.66	2.12 (1.75-2.56)^c^
21-24	23.00	1.23 (1.16-1.30)^c^	10.89	1.36 (1.25-1.47)^c^	4.21	1.20 (1.06-1.35)^e^	2.63	1.52 (1.30-1.78)^c^
≥25	18.77	1 [Reference]	8.02	1 [Reference]	3.53	1 [Reference]	1.73	1 [Reference]
Current age, y^f^								
<21	36.88	2.10 (1.86-2.37)^c^	17.29	1.65 (1.35-2.01)^c^	3.81	0.93 (0.59-1.46)	0.79	0.29 (0.11-0.81)^e^
21-24	32.41	1.84 (1.71-1.99)^c^	14.12	1.34 (1.21-1.50)^c^	5.21	1.27 (1.07-1.52)^g^	1.90	0.70 (0.54-0.91)^g^
25-29	21.79	1.24 (1.17-1.31)^c^	10.97	1.04 (0.97-1.13)	4.44	1.08 (0.96-1.22)	2.38	0.88 (0.76-1.03)
30-34	17.58	1 [Reference]	10.51	1 [Reference]	4.10	1 [Reference]	2.70	1 [Reference]
35-39	16.84	0.96 (0.89-1.03)	9.71	0.92 (0.85-1.00)	4.04	0.99 (0.87-1.12)	3.12	1.16 (1.00-1.34)
≥40	15.73	0.90 (0.80-1.01)	10.30	0.98 (0.86-1.11)	4.21	1.03 (0.85-1.23)	3.29	1.22 (0.98-1.52)
**Family characteristics**								
Marital status								
Military-civilian marriage	23.34	1 [Reference]	10.71	1 [Reference]	4.54	1 [Reference]	2.67	1 [Reference]
Dual-military marriage	25.16	1.08 (1.00-1.17)	17.18	1.61 (1.47-1.76)^c^	4.42	0.97 (0.81-1.17)	3.64	1.36 (1.09-1.70)^g^
Divorced, separated, or widowed	18.87	0.81 (0.74-0.89)^c^	12.65	1.18 (1.07-1.31)^g^	2.74	0.60 (0.50-0.74)^c^	2.76	1.03 (0.83-1.29)
Never married	18.24	0.78 (0.73-0.83)^c^	10.01	0.94 (0.85-1.03)	2.97	0.65 (0.54-0.79)^c^	2.35	0.88 (0.69-1.13)
Children at entry into active duty								
No	23.62	1 [Reference]	11.44	1 [Reference]	4.09	1 [Reference]	2.47	1 [Reference]
Yes	21.16	0.90 (0.86-0.94)^c^	10.53	0.92 (0.86-0.98)^e^	4.66	1.14 (1.03-1.26)^e^	2.99	1.21 (1.07-1.37)^g^
No. of dependent children								
1	16.31	0.65 (0.62-0.68)^c^	6.31	0.55 (0.51-0.59)^c^	2.55	0.58 (0.52-0.65)^c^	0.96	0.42 (0.35-0.50)^c^
2	25.18	1 [Reference]	11.44	1 [Reference]	4.36	1 [Reference]	2.26	1 [Reference]
≥3	41.09	1.63 (1.56-1.71)^c^	21.46	1.88 (1.77-2.00)^c^	7.09	1.63 (1.48-1.79)^c^	5.25	2.32 (2.06-2.61)^c^
Age of youngest child, y								
0-1	28.12	5.46 (4.39-6.78)^c^	12.01	1.31 (1.13-1.52)^c^	3.19	0.80 (0.64-1.01)	1.95	1 [Reference]
2-4	21.93	4.26 (3.43-5.28)^c^	10.80	1.18 (1.02-1.36)^e^	4.55	1.15 (0.92-1.42)	2.75	1.41 (1.23-1.63)^c^
5-12	14.46	2.81 (2.26-3.48)^c^	10.49	1.15 (1.00-1.32)	5.38	1.36 (1.11-1.66)^g^	3.17	1.63 (1.40-1.89)^c^
≥13	5.16	1 [Reference]	9.16	1 [Reference]	3.97	1 [Reference]	3.19	1.64 (1.28-2.11)^c^
**Military-related characteristics**								
Military rank or pay grade								
E1-E3	28.98	1.18 (1.12-1.24)^c^	12.06	0.92 (0.83-1.01)	6.39	1.30 (1.10-1.53)^g^	3.43	1.02 (0.79-1.32)
E4-E6	24.55	1 [Reference]	13.15	1 [Reference]	4.92	1 [Reference]	3.36	1 [Reference]
E7-E9	11.97	0.49 (0.45-0.53)^c^	7.62	0.58 (0.53-0.63)^c^	3.25	0.66 (0.58-0.76)^c^	1.86	0.55 (0.47-0.65)^c^
Warrant officer	12.08	0.49 (0.40-0.60)^c^	7.08	0.54 (0.43-0.67)^c^	3.94	0.80 (0.60-1.06)	1.83	0.54 (0.38-0.79)^g^
O1-O3	9.47	0.39 (0.33-0.45)^c^	6.07	0.46 (0.39-0.55)^c^	2.46	0.50 (0.38-0.66)^c^	1.59	0.47 (0.33-0.67)^c^
≥O4	6.23	0.25 (0.21-0.31)^c^	4.12	0.31 (0.26-0.38)^c^	2.03	0.41 (0.32-0.54)^c^	1.12	0.33 (0.24-0.47)^c^
Deployment status								
Currently deployed	18.07	1 [Reference]	6.97	1 [Reference]	1.77	1 [Reference]	2.11	1 [Reference]
Ever deployed	18.90	1.05 (0.99-1.11)	10.49	1.51 (1.38-1.65)^c^	4.30	2.43 (2.03-2.91)^c^	2.62	1.24 (1.06-1.46)^g^
Never deployed	39.33	2.18 (2.05-2.32)^c^	17.69	2.54 (2.29-2.81)^c^	6.64	3.76 (3.09-4.57)^c^	4.00	1.90 (1.55-2.32)^c^

Abbreviations: E*x*, enlisted pay grade *x*; FM, family-months; OR, odds ratio; O*x*, commissioned officer pay grade *x*; SRE, standardized rate estimate.

^a^ Models included linear spline knots, fiscal years, and calendar months and were adjusted for sponsor race, ethnicity, educational level, and military service branch.

^b^ Calculated assuming other factors were at their samplewide means.

^c^ *P* < .001.

^d^ For nonbiological children, parental age at birth of oldest child was calculated based on the first month of the child’s inclusion in the Active Duty Family data file.

^e^ *P* < .05.

^f^ A time-varying variable, defined as the age of the service member sponsor at each case or at-risk month.

^g^ *P* < .01.

#### Sponsor Sociodemographic Characteristics

In multivariable analysis (Table 3), families of female sponsors had higher odds of every maltreatment type (eg, sexual abuse: OR, 1.39 [95% CI, 1.18-1.64]; physical abuse: OR, 1.82 [95% CI, 1.70-1.95]). Early parenting, indexed by younger sponsor age at the birth of the first child (<21 years), was also associated with higher odds of all maltreatment types (eg, neglect, OR, 1.32 [95% CI, 1.22-1.41]; sexual abuse: OR, 2.12 [95% CI, 1.75-2.56]). In contrast, sponsor age at the time of the at-risk FM or the case FM was associated with some but not all maltreatment types. Younger sponsor age (<21 years) was associated with the highest odds of neglect (OR, 2.10 [95% CI, 1.86-2.37]) and physical abuse (OR, 1.65 [95% CI, 1.35-2.01]). Similarly for emotional abuse, sponsor age of 21 to 24 years was associated with the highest odds (OR, 1.27 [95% CI, 1.07-1.52]). In contrast, for sexual abuse, younger sponsor age (<21 years) was associated with the lowest odds (OR, 0.29 [95% CI, 0.11-0.81]).

#### Family Characteristics

Associations between parental marital status and risk of maltreatment varied across maltreatment types in multivariable analysis (Table 3). Compared with families in which the sponsor was married to a civilian, dual-military marriages were associated with higher odds of physical abuse (OR, 1.61 [95% CI, 1.47-1.76]) and sexual abuse (OR, 1.36 [95% CI, 1.09-1.70]) and families of sponsors who were divorced, separated, or widowed had lower odds of neglect (OR, 0.81 [95% CI, 0.74-0.89]) and emotional abuse (OR, 0.60 [95% CI, 0.50-0.74]) but higher odds of physical abuse (OR, 1.18 [95% CI, 1.07-1.31]). Families of never-married sponsors had lower odds of neglect (OR, 0.78 [95% CI, 0.73-0.83]) and emotional abuse (OR, 0.65 [95% CI, 0.54-0.79]).

Parenting prior to active duty service and the age of the youngest child in the family were also associated with differential risk across the child maltreatment types. Sponsors who had children prior to active duty service had higher odds of emotional abuse (OR, 1.14 [95% CI, 1.03-1.26]) and sexual abuse (OR, 1.21 [95% CI, 1.07-1.37]) but lower odds of neglect (OR, 0.90 [95% CI, 0.86-0.94]) and physical abuse (OR, 0.92 [95% CI, 0.86-0.98]). Compared with families in which the youngest child was an adolescent (age 13-17 years), families in which the youngest child was an infant (age 0-1 year) had higher odds of neglect (OR, 5.46 [95% CI, 4.39-6.78]) and physical abuse (OR, 1.31 [95% CI, 1.13-1.52]). Families in which the youngest child was a toddler (age 2-4 years) also had higher odds of neglect (OR, 4.26 [95% CI, 3.43-5.28]) and physical abuse (OR, 1.18 [95% CI, 1.02-1.36]). Families in which the youngest child was in middle childhood (age 5-12 years) had higher odds of neglect (OR, 2.81 [95% CI, 2.26-3.48]) and emotional abuse (OR, 1.36 [95% CI, 1.11-1.66]). Compared with families in which the youngest child was an infant, families in which the youngest child was a toddler, in middle childhood, and in adolescence had higher odds of sexual abuse (toddler: OR, 1.41 [95% CI, 1.23-1.63]; middle childhood: OR, 1.63 [95% CI, 1.40-1.89]; adolescence: OR, 1.64 [95% CI, 1.28-2.11]).

In contrast, the associations between number of dependent children and risk of child maltreatment were consistent across child maltreatment types. Compared with families with 2 children, families with 1 child had lower odds of each child maltreatment type (eg, sexual abuse: OR, 0.42 [95% CI, 0.35-0.50]; neglect: OR, 0.65 [95% CI, 0.62-0.68]) and families with 3 or more children had higher odds (eg, emotional abuse: OR, 1.63 [95% CI, 95% CI, 1.48-1.79]; sexual abuse: OR, 2.32 [95% CI, 2.06-2.61]).

#### Military-Related Characteristics

Associations between lower military rank and odds of child maltreatment varied across the child maltreatment types in multivariable analysis (Table 3). Compared with families of midlevel enlisted sponsors (enlisted pay grade 4 [E4] to E6), families of junior enlisted (E1 to E3) sponsors had higher odds of neglect (OR, 1.18 [95% CI, 1.12-1.24]) and emotional abuse (OR, 1.30 [95% CI, 1.10-1.53]) but not physical abuse (OR, 0.92 [95% CI, 0.83-1.01]) or sexual abuse (OR, 1.02 [95% CI, 0.79-1.32]). Odds of each child maltreatment type were lower in families of noncommissioned officers (E7 to E9) (eg, neglect: OR, 0.49 [95% CI, 0.45-0.53]; emotional abuse: OR, 0.66 [95% CI, 0.58-0.76]), warrant officers (neglect: OR, 0.49 [95% CI, 0.40-0.60]; physical abuse: OR, 0.54 [95% CI, 0.43-0.67]; sexual abuse: OR, 0.54 [95% CI, 0.38-0.79]), and officer families (eg, neglect: OR, 0.25 [95% CI, 0.21-0.31]; emotional abuse: OR, 0.50 [95% CI, 0.38-0.66]) with the exception of emotional abuse in families of warrant officers (OR, 0.80 [95% CI, 0.60-1.06]).

Associations between deployment status and odds of child maltreatment were relatively consistent across child maltreatment types. Compared with families of currently deployed sponsors, odds of all 4 child maltreatment types were higher in families of never-deployed sponsors (eg, sexual abuse: OR, 1.90 [95% CI, 1.55-2.32]; emotional abuse: OR, 3.76 [95% CI, 3.09-4.57]). Odds of physical abuse (OR, 1.51 [95% CI, 1.38-1.65]), emotional abuse (OR, 2.43 [95% CI, 2.03-2.91]), and sexual abuse (OR, 1.24 [95% CI, 1.06-1.46) were also higher in families of previously deployed sponsors compared with families of sponsors who were deployed at the time of the case FM or at-risk FM.

### Child Age–Related Changes in the Risk of First Occurrences of Child Maltreatment by Type

The Figure illustrates variations in the rates of each maltreatment type for children from birth to age 18 years. Risk of child neglect peaked at month 3 (48.61 per 100 000 FM), remained elevated from 1 to 4 years, and gradually declined over the next 5 years. Linear spline analysis indicated that the slope for first occurrences of neglect increased significantly from months 0 to 3, declined sharply from months 3 to 44, and gradually declined after month 44. Physical abuse also peaked at month 3 (25.49 per 100 000 FM), declined from months 4 to 11, increased from months 12 to 68, and subsequently declined starting at month 69. Risk for sexual abuse increased from months 0 to 42, followed by a nonsignificant increase starting at month 43. Risk of emotional abuse increased from months 0 to 53, declined starting at month 54, and peaked during middle childhood and adolescence (6 per 100 000 FM at months 113 and 143). Risk for sexual abuse and emotional abuse was generally less than 6 per 100 000 FMs across all other ages.

**Figure.**
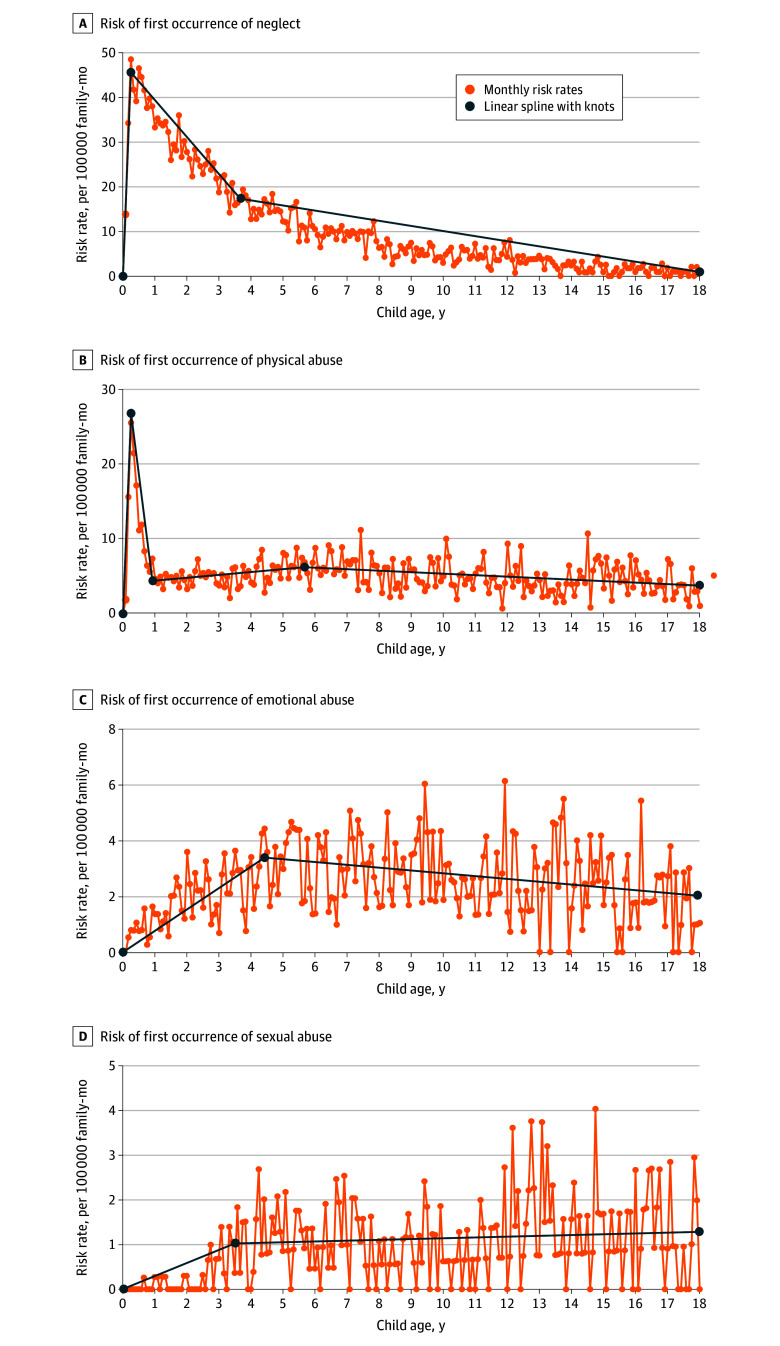
Line Graphs of Risk Rates of First Occurrence of Child Maltreatment by Type Data are for the first biological child born on or after the service member parent entered active duty military service. All rates were adjusted for sampling weights (1.0:1.0 for child maltreatment incident months and 62.5:1.0 for at-risk months). Dark blue lines indicate fitted linear splines and knots.

## Discussion

This retrospective cohort study examined differences in factors associated with first occurrences of 4 child maltreatment types in active duty military families from all service branches and compared changes in risk rates of first occurrences over time. Results revealed both consistency and variation in the parent, family, and military characteristics associated with neglect, physical abuse, emotional abuse, and sexual abuse. In particular, early parenting and a larger number of dependent children were associated with increased risk for all child maltreatment types in multivariable models. These factors have been shown to be associated with increased risk of child maltreatment defined broadly as well as for single maltreatment types in civilian families.^6,7,8^ The present study advances prior research by demonstrating that the risk associated with these factors extended to multiple child maltreatment types in a single population-based sample of military families. The consistency in findings for these factors across all child maltreatment types suggests that families with these characteristics should be prioritized for prevention efforts. However, additional research is needed to elucidate the specific mechanisms through which these characteristics are associated with elevated risk of each child maltreatment type.

Two additional factors associated with increased risk across all child maltreatment types also warrant further investigation: female service member families and never-deployed service member families. The elevated risk observed in female service member families may reflect differences in the experiences of female compared with male service member families, including variations in childcare responsibilities, unique challenges of parenting as a mother while serving in the military, or potential risk associated with characteristics of civilian adult partners, which may in turn contribute to differential risk of child maltreatment. Future studies are needed to clarify the causal drivers of elevated risk in female service member families to inform the development of targeted prevention and intervention strategies.

Our finding that current deployment was associated with decreased risk for child maltreatment types compared with no deployment and previous deployment contrasts with prior reports of increased risk associated with postdeployment compared with current deployment.^22,23^ Differences in study design, deployment measurement, and analytic approaches may account for the discrepant results across studies. Our findings suggest that deployment may be associated with reduced risk for child maltreatment if the deployed service member is the perpetrator of abuse. One possible explanation for our finding that never-deployed status was associated with the highest risk of all child maltreatment types is that this relatively small subgroup of families may include service members who are nondeployable due to medical or psychiatric conditions that are independently associated with increased risk for child maltreatment.^24,25^ These hypotheses require further investigation.

Several other factors, including parental marital status, were differentially associated with the 4 child maltreatment types. Compared with families of service members married to civilians, families of sponsors who were never married and families of sponsors who were divorced, separated, or widowed had lower risk of neglect and emotional abuse. These findings are in contrast to research on civilian samples in which single-parent family structure was generally associated with increased risk of child maltreatment compared with 2-parent households.^6,26^ Our results also showed that dual-military families had higher odds of physical and sexual abuse compared with families of service members married to civilians. Dual-military families may experience more frequent or co-occurring parental deployments, deployment-related mental health conditions, and greater childcare instability, which in turn may be associated with increased risk of harm to their children.^27,28^ Resources that address the unique challenges of dual-military families should be evaluated to determine their ability to remediate risk in dual-military families. Assessing caregiving stability and stress related to dual-military service obligations during health care appointments may improve the identification of at-risk dual-military families and help clinicians refer families to preventive services.

Findings for other factors, including current parental age, youngest child age, and parenting prior to military service, suggest that families have differential risk depending on whether they are in early vs later phases of the family life course. In particular, younger parental age was associated with increased risk of neglect and physical abuse, whereas older parental age was associated with increased risk of sexual abuse. Similarly, families in which the youngest child was aged 4 years or younger had higher odds of neglect and physical abuse, whereas families with older children had higher odds of sexual abuse. In addition, families with children prior to military service had higher odds of emotional and sexual abuse but lower odds of neglect and physical abuse. Collectively, these findings are consistent in pointing to elevated risk of neglect and physical abuse in families with young parents or young children and increased risk of emotional and sexual abuse in more established families at later phases of the family life course. Future investigations of the causal drivers of elevated risk associated with parental age, family composition, and the timing of parenting are needed to inform the development of screening protocols to improve the identification of children at risk of specific child maltreatment types. Greater understanding of how these factors vary across the military family life course may also help practitioners offer resources that are tailored to the needs and challenges of parents at different life stages.

Results regarding changes in the risk of maltreatment across childhood further showed differences in the ages at which children were at greater risk of each maltreatment type. Consistent with research on medical diagnoses of child neglect,^29^ the risk of neglect was highest during the first 3 to 4 years of life, with a peak at month 3. Risk of physical abuse also peaked at month 3 and remained elevated through month 12. The approximate 6-fold increase in risk of physical abuse in early infancy compared with older children is consistent with reports of the age-related incidence of abusive head trauma,^30^ which has been associated with infant fatality in military families.^31^ Together, these results underscore the need for enhanced child maltreatment prevention efforts in military families with infants, especially newborns. In contrast, risk of emotional and sexual abuse emerged in early childhood followed by peaks in middle childhood and adolescence, suggesting the need for enhanced prevention efforts for these abuse types in families with older children.

### Limitations

Several limitations should be considered when interpreting the results. First, the study included child maltreatment incidents that were reported to the Family Advocacy Program and met the DOW definition of abuse or neglect at the time of the incident determination. Results may not generalize to undocumented incidents and reports that did not meet DOW criteria for child maltreatment.^18^ In addition, although our ability to merge multiple DOW administrative datasets on child maltreatment incidents and characteristics of service member families allowed us to examine a broader range of risk factors and confounding variables compared with prior studies, our analyses were limited to data available in administrative records. Failure to control for other potential risk factors, such as parental substance use, mental health disorders, and psychosocial functioning, may have biased the reported associations. Future research is needed to identify the pathways through which certain factors, including female service member families and families of service members without deployments, are associated with higher risk of specific child maltreatment types. Furthermore, our models did not differentiate nondeployable service members from service members who were deployed after the child maltreatment occurrence. In addition, analyses did not statistically compare differences in the magnitude of associations across child maltreatment types or examine factors associated with child maltreatment incidents involving more than 1 child maltreatment type.

## Conclusions

In this cohort study of active duty service member families, families with female service members, never-deployed service members, 3 or more dependent children, and service members who were young when they became parents had increased risk of all child maltreatment types. These results suggest a need to develop prevention strategies tailored to specific high-risk groups as well as dynamic prevention approaches that reflect changes in risk throughout the military family life course to optimize the impact of prevention efforts on children’s risk of harm.

## Notes

Supplement 1. **eAppendix.** Department of War Definitions of Child Maltreatment Types
eReference
**eTable 1.** Sponsor Sociodemographic, Family, and Military-Related Characteristics by Child Maltreatment Type in Active Duty Families, Fiscal Years 2009 to 2018 **eTable 2.** Univariable Analysis of Sponsor Sociodemographic, Family, and Military-Related Characteristics and First Occurrence of Child Maltreatment by Type in Active Duty Families, Fiscal Years 2009 to 2018 **eTable 3.** Multivariable Analysis of Sponsor Sociodemographic, Family, and Military-Related Characteristics and First Occurrence of Child Maltreatment by Type in Active Duty Families, Fiscal Years 2009 to 2018 **eTable 4.** Variance Inflation Factors for Covariates Included in Multivariable Logistic Regressions Predicting Child Maltreatment Types

Supplement 2.
Data Sharing Statement
