# Supplementary material for: Factors Associated With First Occurrences of Child Maltreatment in Military Families
Source: JAMA Netw Open. 2026 May 13;9(5):e2612199. doi: 10.1001/jamanetworkopen.2026.12199 (PMC13173382; doi:10.1001/jamanetworkopen.2026.12199)
Supplement: Supplement 1. — eAppendix. Department of War Definitions of Child Maltreatment Types eReference eTable 1. Sponsor Sociodemographic, Family, and Military-Related Characteristics by Child Maltreatment Type in Active Duty Families, Fiscal Years 2009 to 2018 eTable 2. Univariable Analysis of Sponsor Sociodemographic, Family, and Military-Related Characteristics and First Occurrence of Child Maltreatment by Type in Active Duty Families, Fiscal Years 2009 to 2018 eTable 3. Multivariable Analysis of Sponsor Sociodemographic, Family, and Military-Related Characteristics and First Occurrence of Child Maltreatment by Type in Active Duty Families, Fiscal Years 2009 to 2018 eTable 4. Variance Inflation Factors for Covariates Included in Multivariable Logistic Regressions Predicting Child Maltreatment Types [file jamanetwopen-e2612199-s001.pdf]

Supplemental Online Content

Cozza SJ, Ogle CM, Fisher JE, et al. Factors associated with first occurrences of child maltreatment in military families. *JAMA Netw Open*. 2026;9(5):e2612199.
doi:10.1001/jamanetworkopen.2026.12199

eAppendix. Department of War Definitions of Child Maltreatment Types
eReference
eTable 1. Sponsor Sociodemographic, Family, and Military-Related Characteristics by Child Maltreatment Type in Active Duty Families, Fiscal Years 2009 to 2018
eTable 2. Univariable Analysis of Sponsor Sociodemographic, Family, and Military-Related Characteristics and First Occurrence of Child Maltreatment by Type in Active Duty Families, Fiscal Years 2009 to 2018
eTable 3. Multivariable Analysis of Sponsor Sociodemographic, Family, and Military-Related Characteristics and First Occurrence of Child Maltreatment by Type in Active Duty Families, Fiscal Years 2009 to 2018
eTable 4. Variance Inflation Factors for Covariates Included in Multivariable Logistic Regressions Predicting Child Maltreatment Types

This supplemental material has been provided by the authors to give readers additional information about their work.

© 2026 Cozza SJ et al. *JAMA Network Open*.

eAppendix. Department of War Definitions of Child Maltreatment Types

Child Maltreatment Types. Each child maltreatment type was defined in accordance with Department of War criteria for child abuse and neglect, as outlined below.[1]

Child neglect: The negligent treatment of a child through egregious acts or omissions below the lower bounds of normal caregiving, which shows a striking disregard for the child's well-being, under circumstances indicating that the child's welfare has been harmed or threatened by the deprivation of age-appropriate care.

Child physical abuse: The non-accidental use of physical force such as grabbing, pushing, holding, slapping, choking, punching, kicking, sitting or standing upon, lifting and throwing, burning, immersing in hot liquids or pouring hot liquids upon, hitting with an object (such as a belt or electrical cord), and assaulting with a knife, firearm, or other weapon that causes or may cause significant impact. Does not include discipline administered by a parent or legal guardian to his or her child provided it is reasonable in manner and moderate in degree and otherwise does not constitute cruelty.

Child emotional abuse: A type of child abuse including non-accidental acts resulting in an adverse effect upon the child's psychological well-being. Emotional abuse includes intentional berating, disparaging, or other verbally abusive behavior toward the child, and excessive disciplinary acts that may not cause observable physical injury.

Child sexual abuse: The employment, use, persuasion, inducement, enticement, or coercion of any child to engage in, or assist any other person to engage in, any sexually explicit conduct or simulation of such conduct for the purpose of producing a visual depiction of such conduct; or the rape, and in cases of caretaker or inter-familial relationships, statutory rape, molestation, prostitution, or other form of sexual exploitation of children, or incest with children.

© 2026 Cozza SJ et al. *JAMA Network Open*.

eReference

1. Department of Defense. *DoD Manual 6400.01, Volume 3: Family Advocacy Program: Clinical Case Staff Meeting and Incident Determination Committee.* August 11, 2016. Incorporating Change 1, effective July 16, 2021. Accessed 02/03/2026.

© 2026 Cozza SJ et al. *JAMA Network Open*.

eTable 1. Sponsor Sociodemographic, Family, and Military-Related Characteristics by Child Maltreatment Type in Active Duty Families, Fiscal Years 2009 to 2018

	Neglect		Physical Abuse		Emotional Abuse		Sexual Abuse	
	Cases n (%)	Total[a] n (%)	Cases n (%)	Total n (%)	Cases n (%)	Total n (%)	Cases n (%)	Total n (%)
**I. Sponsor sociodemographic characteristics**								
Sex								
Female	2,145 (14.86)	6,108,708 (9.38)	1,273 (17.82)	6,107,836 (9.38)	395 (14.34)	6,106,958 (9.38)	190 (10.98)	6,106,753 (9.38)
Male	12,288 (85.14)	59,019,288 (90.62)	5,872 (82.18)	59,012,872 (90.62)	2,360 (85.66)	59,009,360 (90.62)	1,541 (89.02)	59,008,541 (90.62)
Age at birth of oldest child (in years)[b]								
< 21	5,920 (41.29)	15,712,858 (24.39)	2,991 (42.45)	15,709,929 (24.39)	1,121 (40.97)	15,708,059 (24.39)	788 (45.76)	15,707,726 (24.39)
21 − 24	5,671 (39.55)	21,627,296 (33.57)	2,600 (36.72)	21,624,225 (33.57)	954 (34.87)	21,622,579 (33.57)	597 (34.67)	21,622,222 (33.57)
≥ 25	2,748 (19.16)	27,084,311 (42.04)	1,489 (21.03)	27,083,052 (42.04)	661 (24.16)	27,082,224 (42.04)	337 (19.57)	27,081,900 (42.04)
Current age (in years)								
< 21	638 (4.42)	773,951 (1.20)	184 (2.58)	773,497 (1.20)	25 (0.91)	773,338 (1.20)	5 (0.29)	773,318 (1.20)
21 − 24	4,555 (31.56)	7,147,993 (11.05)	1,279 (17.91)	7,144,717 (11.04)	370 (13.44)	7,143,808 (11.04)	106 (6.12)	7,143,544 (11.04)
25 − 29	4,819 (33.39)	15,131,757 (23.38)	2,002 (28.03)	15,128,940 (23.38)	742 (26.94)	15,127,680 (23.38)	397 (22.93)	15,127,335 (23.38)
30 − 34	2,616 (18.13)	15,829,741 (24.46)	1,811 (25.35)	15,828,936 (24.46)	724 (26.29)	15,827,849 (24.46)	520 (30.04)	15,827,645 (24.46)
35 − 39	1,281 (8.88)	13,968,844 (21.58)	1,216 (17.02)	13,968,779 (21.59)	559 (20.30)	13,968,122 (21.59)	462 (26.69)	13,968,025 (21.59)

© 2026 Cozza SJ et al. *JAMA Network Open*.

≥ 40	522 (3.62)	11,863,897 (18.33)	651 (9.11)	11,864,026 (18.33)	334 (12.13)	11,863,709 (18.34)	241 (13.92)	11,863,616 (18.34)
Race[c]								
Asian	296 (2.06)	2,385,859 (3.70)	200 (2.81)	2,385,763 (3.70)	85 (3.10)	2,385,648 (3.70)	48 (2.78)	2,385,611 (3.70)
Black	3,660 (25.41)	12,167,785 (18.89)	2,123 (29.84)	12,166,248 (18.89)	643 (23.42)	12,164,768 (18.89)	302 (17.51)	12,164,427 (18.89)
Native American	252 (1.75)	884,815 (1.37)	115 (1.62)	884,678 (1.37)	61 (2.22)	884,624 (1.37)	29 (1.68)	884,592 (1.37)
Pacific Islander	122 (0.85)	591,247 (0.92)	75 (1.05)	591,200 (0.92)	36 (1.31)	591,161 (0.92)	15 (0.87)	591,140 (0.92)
White	9,291 (64.51)	43,733,229 (67.90)	4,091 (57.51)	43,728,029 (67.90)	1,711 (62.31)	43,725,649 (67.90)	1,191 (69.04)	43,725,129 (67.90)
Other[d]	782 (5.43)	4,642,720 (7.21)	510 (7.17)	4,642,448 (7.21)	210 (7.65)	4,642,148 (7.21)	140 (8.12)	4,642,078 (7.21)
Ethnicity[c]								
Non-Hispanic	12,522 (88.11)	55,697,085 (87.46)	6162 (87.75)	55,690,725 (87.46)	2,339 (85.87)	55,686,902 (87.46)	1,480 (86.60)	55,686,043 (87.46)
Hispanic	1,689 (11.89)	7,987,439 (12.54)	860 (12.25)	7,986,610 (12.54)	385 (14.13)	7,986,135 (12.54)	229 (13.40)	7,985,979 (12.54)
Education level								
Less than High School	97 (0.68)	282,097 (0.44)	29 (0.41)	282,029 (0.44)	14 (0.51)	282,014 (0.44)	15 (0.88)	282,015 (0.44)
High School or equivalent	12,046 (84.02)	36,978,109 (58.18)	5,251 (74.32)	36,971,314 (58.18)	1900 (69.85)	36,967,963 (58.18)	1,221 (71.57)	36,967,284 (58.18)
Some college	1,427 (9.95)	10,259,365 (16.14)	1,086 (15.37)	10,259,024 (16.14)	475 (17.46)	10,258,413 (16.14)	276 (16.18)	10,258,214 (16.14)
College degree or higher	767 (5.35)	16,034,580 (25.23)	699 (9.89)	16,034,512 (25.23)	331 (12.71)	16,034,144 (25.23)	194 (11.37)	16,034,007 (25.23)

© 2026 Cozza SJ et al. *JAMA Network Open*.

**II. Family characteristics**								
Marital status								
Military-civilian marriage	11,888 (82.38)	53,784,451 (82.96)	5,519 (77.26)	53,778,082 (82.96)	2,378 (86.35)	53,774,941 (82.96)	1,473 (85.10)	53,774,036 (82.96)
Dual-military marriage	729 (5.05)	3,287,354 (5.07)	589 (8.25)	3,287,214 (5.07)	134 (4.87)	3,286,759 (5.07)	88 (5.08)	3,286,713 (5.07)
Divorced/Separated/ Widowed	497 (3.44)	3,388,497 (5.23)	439 (6.15)	3,388,439 (5.23)	113 (4.10)	3,388,113 (5.23)	91 (5.26)	3,388,091 (5.23)
Never married	1,317 (9.13)	4,370,005 (6.74)	596 (8.34)	4,369,284 (6.74)	129 (4.68)	4,368,817 (6.74)	79 (4.56)	4,368,767 (6.74)
Children at entry into active duty								
No	9,075 (63.12)	47,670,013 (73.38)	4,430 (62.18)	47,665,368 (73.38)	1,594 (57.94)	47,662,532 (73.38)	885 (51.13)	47,661,823 (73.38)
Yes	5,303 (36.88)	17,293,428 (26.62)	2,694 (37.82)	17,290,819 (26.62)	1,157 (42.06)	17,289,282 (26.62)	846 (48.87)	17,288,971 (26.62)
Number of dependent children								
1	5,835 (40.43)	24,570,210 (37.73)	1,954 (27.35)	24,566,329 (37.73)	646 (23.45)	24,565,021 (37.73)	212 (12.25)	24,564,587 (37.73)
2	4,596 (31.84)	23,572,784 (36.20)	2,310 (32.33)	23,570,498 (36.20)	994 (36.08)	23,569,182 (36.20)	530 (30.62)	23,568,718 (36.20)
3 or more	4,002 (27.73)	16,983,752 (26.08)	2,881 (40.32)	16,982,631 (26.08)	1,115 (40.47)	16,980,865 (26.08)	989 (57.13)	16,980,739 (26.08)
Age of youngest child								
0 - 1	7,952 (55.10)	20,878,452 (32.14)	2,805 (39.29)	20,873,305 (32.14)	687 (24.95)	20,871,187 (32.14)	353 (20.42)	20,870,853 (32.14)
2 - 4	4,446 (30.80)	18,412,446 (28.35)	2,108 (29.53)	18,410,108 (28.35)	926 (33.62)	18,408,926 (28.35)	544 (31.46)	18,408,544 (28.35)
5 - 12	1,936 (13.41)	20,643,561 (31.78)	1,956 (27.40)	20,643,581 (31.79)	1,014 (36.82)	20,642,639 (31.79)	714 (41.30)	20,642,339 (31.79)
≥ 13	99 (0.69)	5,016,662 (7.72)	270 (3.78)	5,016,833 (7.72)	127 (4.61)	5,016,690 (7.73)	118 (6.82)	5,016,681 (7.73)

© 2026 Cozza SJ et al. *JAMA Network Open*.

**III. Military-related characteristics**								
Service branch								
Navy	2260 (15.66)	14,573,760 (22.38)	1,603 (22.44)	14,573,103 (22.38)	462 (16.77)	14,571,962 (22.38)	473 (27.33)	14,571,973 (22.38)
Army	7,904 (54.76)	28,132,529 (43.20)	3,240 (45.35)	28,127,865 (43.19)	1,214 (44.07)	28,125,839 (43.19)	876 (50.61)	28,125,501 (43.19)
Air Force	2,350 (16.28)	15,379,725 (23.61)	1,553 (21.74)	15,378,928 (23.62)	676 (24.54)	15,378,051 (23.62)	266 (15.37)	15,377,641 (23.62)
Marine Corps	1,919 (13.30)	7,042,294 (10.81)	749 (10.48)	7,041,124 (10.81)	403 (14.63)	7,040,778 (10.81)	116 (6.70)	7,040,491 (10.81)
Military rank								
E1 to E3	2909 (20.16)	4,549,284 (6.99)	785 (10.99)	4,547,160 (6.98)	257 (9.33)	4,546,632 (6.98)	94 (5.43)	4,546,469 (6.98)
E4 to E6	10,289 (71.29)	35,102,789 (53.90)	5,061 (70.83)	35,097,561 (53.90)	1,855 (67.33)	35,094,355 (53.89)	1,213 (70.08)	35,093,713 (53.89)
E7 to E9	697 (4.83)	11,422,760 (17.54)	797 (11.15)	11,422,860 (17.54)	384 (13.94)	11,422,447 (17.54)	269 (15.54)	11,422,332 (17.54)
Warrant officer	108 (0.75)	1,619,921 (2.49)	94 (1.32)	1,619,907 (2.49)	59 (2.14)	1,619,872 (2.49)	32 (1.85)	1,619,845 (2.49)
O1 to O3	281 (1.95)	5,221,406 (8.02)	229 (3.21)	5,221,354 (8.02)	89 (3.23)	5,221,214 (8.02)	60 (3.47)	5,221,185 (8.02)
O4 and higher	149 (1.03)	7,212,149 (11.07)	179 (2.51)	7,212,179 (11.08)	111 (4.03)	7,212,111 (11.08)	63 (3.64)	7,212,063 (11.08)
Deployment status								
Currently deployed	1,521 (10.54)	7,874,459 (12.09)	574 (8.03)	7,873,512 (12.09)	132 (4.79)	7,873,070 (12.09)	176 (10.17)	7,873,114 (12.09)
Ever deployed	8,161 (56.54)	48,317,661 (74.18)	4,898 (68.55)	48,314,398 (74.19)	2,037 (73.94)	48,311,537 (74.19)	1,281 (74.00)	48,310,781 (74.19)
Never deployed	4,751 (32.92)	8,936,439 (13.72)	1,673 (23.41)	8,933,361 (13.72)	586 (21.27)	8,932,274 (13.72)	274 (15.83)	8,931,962 (13.72)

© 2026 Cozza SJ et al. *JAMA Network Open*.

[a]Total includes case and control family-months.
[b]Parental age at birth of oldest child was calculated for non-biological children based on the first month of the child's inclusion in the Active Duty Family data file.
[c]Race and ethnicity were drawn from the Active Duty Military Personnel Master data file. Categories correspond to those in the Active Duty Military Personnel Master data file.
[d]Other includes “Multiple race” and “Unspecified.” “Multiple race” included service members with documented membership in more than one race category.

© 2026 Cozza SJ et al. *JAMA Network Open*.

eTable 2. Univariable Analysis of Sponsor Sociodemographic, Family, and Military-Related Characteristics and First Occurrence of Child Maltreatment by Type in Active Duty Families, Fiscal Years 2009 to 2018

	Neglect		Physical Abuse		Emotional Abuse		Sexual Abuse	
	Rate[a]	Odds Ratio [95% CI]	Rate	Odds Ratio [95% CI]	Rate	Odds Ratio [95% CI]	Rate	Odds Ratio [95% CI]
**I. Sponsor socio-demographic characteristics**								
Sex								
Female	35.11	1.69 [1.61, 1.77]***	20.84	2.10 [1.97, 2.23]***	6.47	1.62 [1.45, 1.80]***	3.11	1.19 [1.03, 1.39]*
Male	20.82	Ref	9.95	Ref	4.00	Ref	2.61	Ref
**χ^2 (df)**		499.06 (1)***		572.91 (1)***		78.21 (1)***		5.19 (1)*
Age at birth of oldest child (in years)[b]								
< 21	37.68	3.71 [3.55, 3.89]***	19.04	3.46 [3.26, 3.69]***	7.14	2.92 [2.66, 3.22]***	5.02	4.03 [3.55, 4.58]***
21 − 24	26.22	2.58 [2.47, 2.70]***	12.02	2.19 [2.05, 2.33]***	4.41	1.81 [1.64, 2.00]***	2.76	2.22 [1.94, 2.54]***
≥ 25	10.15	Ref	5.50	Ref	2.44	Ref	1.24	Ref
**χ^2 (df)**		3,240.39 (2)***		1,537.71 (2)***		483.03 (2)***		471.64 (2)***
Current age (in years)								
< 21	82.43	4.99 [4.58, 5.44]***	23.79	2.08 [1.79, 2.42]***	3.23	0.71 [0.47, 1.05]	0.65	0.20 [0.08, 0.48]***
21 − 24	63.72	3.86 [3.68, 4.05]***	17.90	1.57 [1.46, 1.68]***	5.18	1.13 [1.00, 1.28]	1.48	0.45 [0.37, 0.56]***
25 − 29	31.85	1.93 [1.84, 2.02]***	13.23	1.16 [1.09, 1.23]***	4.90	1.07 [0.97, 1.19]	2.62	0.80 [0.70, 0.91]***
30 − 34	16.53	Ref	11.44	Ref	4.57	Ref	3.29	Ref
35 − 39	9.17	0.56 [0.52, 0.59]***	8.71	0.76 [0.71, 0.82]***	4.00	0.88 [0.78, 0.98]*	3.31	1.01 [0.89, 1.14]
≥ 40	4.40	0.27 [0.24, 0.29]***	5.49	0.48 [0.44, 0.53]***	2.82	0.62 [0.54, 0.70]***	2.03	0.62 [0.53, 0.72]***
**χ^2 (df)**		7,904.0 (5)***		834.75 (5)***		93.08 (5)***		105.89 (5)***
Race[c]								
Asian	12.41	0.58 [0.52, 0.66]***	8.38	0.90 [0.78, 1.03]	3.56	0.91 [0.73, 1.13]	2.01	0.74 [0.55, 0.99]*
Black	30.08	1.42 [1.36, 1.47]***	17.45	1.87 [1.77, 1.97]***	5.29	1.35 [1.23, 1.48]***	2.48	0.91 [0.80, 1.03]
Native American	28.48	1.34 [1.18, 1.52]***	13.00	1.39 [1.15, 1.67]***	6.90	1.76 [1.37, 2.28]***	3.28	1.20 [0.83, 1.74]

© 2026 Cozza SJ et al. *JAMA Network Open*.

Pacific Islander	20.63	0.97 [0.81, 1.16]	12.69	1.36 [1.08, 1.70]**	6.09	1.56 [1.12, 2.17]**	2.54	0.93 [0.56, 1.55]
White	21.24	Ref	9.36	Ref	3.91	Ref	2.72	Ref
Other[d]	16.84	0.79 [0.74, 0.85]***	10.99	1.17 [1.07, 1.29]***	4.52	1.16 [1.00, 1.33]*	3.02	1.11 [0.93, 1.32]
**χ² (df)**		522.47 (5)***		566.94 (5)***		64.11 (5)***		9.18 (5)
Ethnicity[c]								
Non-Hispanic	22.48	Ref	11.06	Ref	4.20	Ref	2.66	Ref
Hispanic	21.15	0.94 [0.89, 0.99]*	10.77	0.97 [0.91, 1.05]	4.82	1.15 [1.03, 1.28]*	2.87	1.08 [0.94, 1.24]
**χ² (df)**		5.59 (1)*		0.55 (1)		6.28 (1)*		1.14 (1)
Education level								
Less than High School	34.39	7.19 [5.82, 8.88]***	10.28	2.36 [1.63, 3.42]***	4.96	2.40 [1.41, 4.10]**	5.32	4.40 [2.60, 7.44]***
High School or equivalent	32.58	6.81 [6.33, 7.33]***	14.20	3.26 [3.01, 3.53]***	5.14	2.49 [2.22, 2.80]***	3.30	2.73 [2.35, 3.18]***
Some college	13.91	2.91 [2.66, 3.17]***	10.59	2.43 [2.21, 2.67]***	4.63	2.24 [1.95, 2.58]***	2.69	2.22 [1.85, 2.67]***
College degree or higher	4.78	Ref	4.36	Ref	2.06	Ref	1.21	Ref
**χ² (df)**		3,356.06 (3)***		883.54 (3)***		234.99 (3)***		175.63 (3)***
**II. Family characteristics**								
Marital status								
Military-civilian marriage	22.10	Ref	10.26	Ref	4.42	Ref	2.74	Ref
Dual-military marriage	22.18	1.00 [0.93, 1.08]	17.92	1.75 [1.60, 1.90]***	4.08	0.92 [0.78, 1.10]	2.68	0.98 [0.79, 1.21]

© 2026 Cozza SJ et al. *JAMA Network Open*.

Divorced/ Separated/ Widowed	14.67	0.66 [0.61, 0.73]***	12.96	1.26 [1.15, 1.39]***	3.34	0.75 [0.62, 0.91]**	2.69	0.98 [0.79, 1.21]
Never married	30.14	1.36 [1.29, 1.44]***	13.64	1.33 [1.22, 1.45]***	2.95	0.67 [0.56, 0.80]***	1.81	0.66 [0.53, 0.83]***
**χ² (df)**		207.27 (3)***		204.66 (3)***		27.65 (3)***		12.93 (3)**
Children at entry into active duty								
No	19.04	Ref	9.29	Ref	3.34	Ref	1.86	Ref
Yes	30.66	1.61 [1.56, 1.67]***	15.58	1.68 [1.60, 1.76]***	6.69	2.00 [1.86, 2.16]***	4.89	2.64 [2.40, 2.90]***
**χ² (df)**		760.86 (1)***		447.24 (1)***		322.60 (1)***		406.44 (1)***
Number of dependent children								
1	23.75	1.22 [1.17, 1.27]***	7.95	0.81 [0.76, 0.86]***	2.63	0.62 [0.57, 0.69]***	0.86	0.38 [0.33, 0.45]***
2	19.50	Ref	9.80	Ref	4.22	Ref	2.25	Ref
3 or more	23.56	1.21 [1.16, 1.26]***	16.96	1.73 [1.64, 1.83]***	6.57	1.56 [1.43, 1.70]***	5.82	2.59 [2.33, 2.88]***
**χ² (df)**		118.12 (2)***		760.11 (2)***		349.79 (2)***		775.37 (2)***
Age of youngest child								
0 - 1	38.09	19.30 [15.83, 23.53]***	13.44	2.50 [2.20, 2.83]***	3.29	1.30 [1.08, 1.57]**	1.69	Ref
2 - 4	24.15	12.24 [10.03, 14.93]***	11.45	2.13 [1.87, 2.42]***	5.03	1.99 [1.65, 2.39]***	2.96	1.75 [1.53, 2.00]***
5 - 12	9.38	4.75 [3.88, 5.82]***	9.48	1.76 [1.55, 2.00]***	4.91	1.94 [1.61, 2.33]***	3.46	2.05 [1.80, 2.32]***
≥ 13	1.97	Ref	5.38	Ref	2.53	Ref	2.35	1.39 [1.13, 1.71]**
**χ² (df)**		3,839.66 (3)***		293.70 (3)***		125.14 (3)***		126.56 (3)***
**III. Military-related characteristics**								
Military service branch								
Navy	15.51	0.55 [0.53, 0.58]***	11.00	0.96 [0.90, 1.01]	3.17	0.74 [0.66, 0.82]***	3.25	1.04 [0.93, 1.17]
Army	28.10	Ref	11.52	Ref	4.32	Ref	3.11	Ref
Air Force	15.28	0.54 [0.52, 0.57]***	10.10	0.88 [0.83, 0.93]***	4.40	1.02 [0.93, 1.12]	1.73	0.56 [0.48, 0.64]***
Marine Corps	27.25	0.97 [0.92, 1.02]	10.64	0.92 [0.85, 1.00]*	5.72	1.33 [1.19, 1.48]***	1.65	0.53 [0.44, 0.64]***

© 2026 Cozza SJ et al. *JAMA Network Open*.

**χ² (df)**		1,115.93 (3)***		19.08 (3)***		76.21 (3)***		114.18 (3)***
Military rank								
E1 to E3	63.94	2.18 [2.09, 2.27]***	17.26	1.20 [1.11, 1.29]***	5.65	1.07 [0.94, 1.22]	2.07	0.60 [0.49, 0.74]***
E4 to E6	29.31	Ref	14.42	Ref	5.29	Ref	3.46	Ref
E7 to E9	6.10	0.21 [0.19, 0.23]***	6.98	0.48 [0.45, 0.52]***	3.36	0.64 [0.57, 0.71]***	2.36	0.68 [0.60, 0.78]***
Warrant officer	6.67	0.23 [0.19, 0.28]***	5.80	0.40 [0.33, 0.49]***	3.64	0.69 [0.53, 0.89]**	1.98	0.57 [0.40, 0.81]**
O1 to O3	5.38	0.18 [0.16, 0.21]***	4.39	0.30 [0.27, 0.35]***	1.70	0.32 [0.26, 0.40]***	1.15	0.33 [0.26, 0.43]***
O4 and higher	2.07	0.07 [0.06, 0.08]***	2.48	0.17 [0.15, 0.20]***	1.54	0.29 [0.24, 0.35]***	0.87	0.25 [0.20, 0.33]***
**χ² (df)**		5,578.96 (5)***		1,229.52 (5)***		308.09 (5)***		206.44 (5)***
Deployment status								
Currently deployed	19.32	Ref	7.29	Ref	1.68	Ref	2.24	Ref
Ever deployed	16.89	0.87 [0.83, 0.92]***	10.14	1.39 [1.28, 1.52]***	4.22	2.51 [2.11, 3.00]***	2.65	1.19 [1.01, 1.39]*
Never deployed	53.16	2.75 [2.60, 2.92]***	18.73	2.57 [2.34, 2.83]***	6.56	3.91 [3.24, 4.72]***	3.07	1.37 [1.14, 1.66]**
**χ² (df)**		4,061.94 (2)***		594.66 (2)***		219.82 (2)***		10.89 (2)**

[a] Rates were calculated per 100,000 family-months.
[b] For non-biological children, age at birth of oldest child was calculated based on the first month of the child's appearance in the Active Duty Family data file.
[c] Race and ethnicity were drawn from the Active Duty Military Personnel Master data files. Race and ethnicity categories correspond to categories in the Active Duty Military Personnel Master data file.
[d] Other includes "Multiple race" and "Unspecified." "Multiple race" included service members with documented membership in more than one race category.
*$p < 0.05$, **$p < 0.01$, ***$p < 0.001$.

© 2026 Cozza SJ et al. *JAMA Network Open*.

eTable 3. Multivariable Analysis of Sponsor Sociodemographic, Family, and Military-Related Characteristics and First Occurrence of Child Maltreatment by Type in Active Duty Families, Fiscal Years 2009 to 2018

	Neglect		Physical Abuse		Emotional Abuse		Sexual Abuse	
	**SRE[a]**	**Odds Ratio [95% CI]**	**SRE**	**Odds Ratio [95% CI]**	**SRE**	**Odds Ratio [95% CI]**	**SRE**	**Odds Ratio [95% CI]**
**I. Sponsor socio-demographic characteristics**								
Sex								
Female	34.78	1.63 [1.55, 1.71]***	18.58	1.82 [1.70, 1.95]***	7.15	1.77 [1.57, 1.99]***	3.63	1.39 [1.18, 1.64]***
Male	21.39	Ref	10.21	Ref	4.05	Ref	2.62	Ref
**χ² (df)**		357.36 (1)***		299.04 (1)***		90.91 (1)***		14.92 (1)***
Age at birth of oldest child (in years)[b]								
< 21	24.67	1.32 [1.22, 1.41]***	13.93	1.74 [1.58, 1.92]***	5.09	1.44 [1.24, 1.68]***	3.66	2.12 [1.75, 2.56]***
21 − 24	23.00	1.23 [1.16, 1.30]***	10.89	1.36 [1.25, 1.47]***	4.21	1.20 [1.06, 1.35]*	2.63	1.52 [1.30, 1.78]***
≥ 25	18.77	Ref	8.02	Ref	3.53	Ref	1.73	Ref
**χ² (df)**		58.68 (2)***		123.81 (2)***		22.96 (2)***		59.61 (2)***
Current age (in years)								
< 21	36.88	2.10 [1.86, 2.37]***	17.29	1.65 [1.35, 2.01]***	3.81	0.93 [0.59, 1.46]	0.79	0.29 [0.11, 0.81]*
21 − 24	32.41	1.84 [1.71, 1.99]***	14.12	1.34 [1.21, 1.50]***	5.21	1.27 [1.07, 1.52]**	1.90	0.70 [0.54, 0.91]**
25 − 29	21.79	1.24 [1.17, 1.31]***	10.97	1.04 [0.97, 1.13]	4.44	1.08 [0.96, 1.22]	2.38	0.88 [0.76, 1.03]
30 − 34	17.58	Ref	10.51	Ref	4.10	Ref	2.70	Ref
35 − 39	16.84	0.96 [0.89, 1.03]	9.71	0.92 [0.85, 1.00]	4.04	0.99 [0.87, 1.12]	3.12	1.16 [1.00, 1.34]
≥ 40	15.73	0.90 [0.80, 1.01]	10.30	0.98 [0.86, 1.11]	4.21	1.03 [0.85, 1.23]	3.29	1.22 [0.98, 1.52]
**χ² (df)**		307.64 (5)***		50.78 (5)***		9.74 (5)		15.78 (5)**
Race[c]								
Asian	15.50	0.71 [0.63, 0.80]***	9.74	0.98 [0.85, 1.14]	4.19	1.03 [0.82, 1.28]	2.08	0.69 [0.52, 0.93]*
Black	26.35	1.21 [1.17, 1.27]***	14.42	1.46 [1.38, 1.54]***	4.81	1.18 [1.07, 1.30]***	1.90	0.63 [0.55, 0.72]***
Native American	25.26	1.16 [1.02, 1.33]*	10.86	1.10 [0.91, 1.33]	6.53	1.60 [1.23, 2.09]***	2.74	0.91 [0.63, 1.33]

© 2026 Cozza SJ et al. *JAMA Network Open*.

Pacific Islander	17.59	0.81 [0.68, 0.97]*	10.24	1.03 [0.82, 1.31]	4.84	1.19 [0.84, 1.67]	2.10	0.70 [0.41, 1.19]
White	21.70	Ref	9.90	Ref	4.08	Ref	3.00	Ref
Other[d]	22.81	1.05 [0.97, 1.14]	11.47	1.16 [1.05, 1.28]**	4.49	1.10 [0.94, 1.29]	3.17	1.06 [0.87, 1.28]
**χ² (df)**		140.81 (5)***		177.16 (5)***		21.84 (5)***		51.05 (5)***
Ethnicity[c]								
Non-Hispanic	23.33	Ref	11.30	Ref	4.29	Ref	2.77	Ref
Hispanic	18.46	0.79 [0.75, 0.84]***	9.73	0.86 [0.80, 0.93]***	4.30	1.00 [0.89, 1.13]	2.24	0.81 [0.69, 0.94]**
**χ² (df)**		71.47 (1)***		13.70 (1)***		0.00 (1)		7.28 (1)**
Education level								
Less than High School	29.21	2.27 [1.81, 2.85]***	8.72	1.06 [0.72, 1.56]	4.81	1.50 [0.85, 2.66]	3.63	1.90 [1.09, 3.31]*
High School or equivalent	24.27	1.89 [1.70, 2.09]***	11.70	1.42 [1.27, 1.60]***	4.59	1.44 [1.21, 1.71]***	2.95	1.54 [1.23, 1.94]***
Some college	17.79	1.38 [1.24, 1.55]***	10.39	1.26 [1.12, 1.43]***	4.12	1.29 [1.07, 1.55]**	2.43	1.27 [1.00, 1.62]*
College degree or higher	12.88	Ref	8.22	Ref	3.20	Ref	1.91	Ref
**χ² (df)**		221.55 (3)***		42.11 (3)***		17.48 (3)***		18.52 (3)***
**II. Family characteristics**								
Marital status								
Military-civilian marriage	23.34	Ref	10.71	Ref	4.54	Ref	2.67	Ref
Dual-military marriage	25.16	1.08 [1.00, 1.17]	17.18	1.61 [1.47, 1.76]***	4.42	0.97 [0.81, 1.17]	3.64	1.36 [1.09, 1.70]**

© 2026 Cozza SJ et al. *JAMA Network Open*.

Divorced/ Separated/ Widowed	18.87	0.81 [0.74, 0.89]***	12.65	1.18 [1.07, 1.31]**	2.74	0.60 [0.50, 0.74]***	2.76	1.03 [0.83, 1.29]
Never married	18.24	0.78 [0.73, 0.83]***	10.01	0.94 [0.85, 1.03]	2.97	0.65 [0.54, 0.79]***	2.35	0.88 [0.69, 1.13]
**χ² (df)**		83.24 (3)***		115.50 (3)***		41.17 (3)***		8.90 (3)*
Children at entry into active duty								
No	23.62	Ref	11.44	Ref	4.09	Ref	2.47	Ref
Yes	21.16	0.90 [0.86, 0.94]***	10.53	0.92 [0.86, 0.98]*	4.66	1.14 [1.03, 1.26]*	2.99	1.21 [1.07, 1.37]**
**χ² (df)**		21.94 (1)***		6.51 (1)*		6.38 (1)*		8.71 (1)**
Number of dependent children								
1	16.31	0.65 [0.62, 0.68]***	6.31	0.55 [0.51, 0.59]***	2.55	0.58 [0.52, 0.65]***	0.96	0.42 [0.35, 0.50]***
2	25.18	Ref	11.44	Ref	4.36	Ref	2.26	Ref
3 or more	41.09	1.63 [1.56, 1.71]***	21.46	1.88 [1.77, 2.00]***	7.09	1.63 [1.48, 1.79]***	5.25	2.32 [2.06, 2.61]***
**χ² (df)**		967.84 (2)***		904.48 (2)*		245.85 (2)***		385.93 (2)***
Age of youngest child								
0 − 1	28.12	5.46 [4.39, 6.78]***	12.01	1.31 [1.13, 1.52]***	3.19	0.80 [0.64, 1.01]	1.95	Ref
2 − 4	21.93	4.26 [3.43, 5.28]***	10.80	1.18 [1.02, 1.36]*	4.55	1.15 [0.92, 1.42]	2.75	1.41 [1.23, 1.63]***
5 − 12	14.46	2.81 [2.26, 3.48]***	10.49	1.15 [1.00, 1.32]	5.38	1.36 [1.11, 1.66]**	3.17	1.63 [1.40, 1.89]***
≥ 13	5.16	Ref	9.16	Ref	3.97	Ref	3.19	1.64 [1.28, 2.11]***
**χ² (df)**		595.00 (3)***		21.14 (3)***		82.70 (3)***		41.48 (3)***
**III. Military-related characteristics**								
Military service branch								
Navy	16.52	Ref	11.58	Ref	3.23	Ref	3.51	Ref
Army	27.19	1.65 [1.56, 1.73]***	10.98	0.95 [0.89, 1.01]	4.12	1.27 [1.14, 1.43]***	2.92	0.83 [0.74, 0.94]**
Air Force	18.35	1.11 [1.05, 1.18]**	11.11	0.96 [0.89, 1.03]	4.97	1.54 [1.35, 1.75]***	1.88	0.54 [0.46, 0.63]***
Marine Corps	22.50	1.36 [1.28, 1.45]***	10.54	0.91 [0.83, 1.00]*	5.91	1.83 [1.59, 2.11]***	1.85	0.53 [0.43, 0.65]***
**χ² (df)**		505.60 (3)***		4.59 (3)		83.85 (3)***		78.87 (3)***

© 2026 Cozza SJ et al. *JAMA Network Open*.

Military rank								
E1 to E3	28.98	1.18 [1.12, 1.24]***	12.06	0.92 [0.83, 1.01]	6.39	1.30 [1.10, 1.53]**	3.43	1.02 [0.79, 1.32]
E4 to E6	24.55	Ref	13.15	Ref	4.92	Ref	3.36	Ref
E7 to E9	11.97	0.49 [0.45, 0.53]***	7.62	0.58 [0.53, 0.63]***	3.25	0.66 [0.58, 0.76]***	1.86	0.55 [0.47, 0.65]***
Warrant officer	12.08	0.49 [0.40, 0.60]***	7.08	0.54 [0.43, 0.67]***	3.94	0.80 [0.60, 1.06]	1.83	0.54 [0.38, 0.79]**
O1 to O3	9.47	0.39 [0.33, 0.45]***	6.07	0.46 [0.39, 0.55]***	2.46	0.50 [0.38, 0.66]***	1.59	0.47 [0.33, 0.67]***
O4 and higher	6.23	0.25 [0.21, 0.31]***	4.12	0.31 [0.26, 0.38]***	2.03	0.41 [0.32, 0.54]***	1.12	0.33 [0.24, 0.47]***
**χ^2 (df)**		470.29 (5)***		251.13 (5)***		75.74 (5)***		79.71 (5)***
Deployment status								
Currently deployed	18.07	Ref	6.97	Ref	1.77	Ref	2.11	Ref
Ever deployed	18.90	1.05 [0.99, 1.11]	10.49	1.51 [1.38, 1.65]***	4.30	2.43 [2.03, 2.91]***	2.62	1.24 [1.06, 1.46]**
Never deployed	39.33	2.18 [2.05, 2.32]***	17.69	2.54 [2.29, 2.81]***	6.64	3.76 [3.09, 4.57]***	4.00	1.90 [1.55, 2.32]***
**χ^2 (df)**		1286.33 (2)***		395.61 (2)***		183.68 (2)***		45.07 (2)***

Note. Models included linear spline knots, fiscal years, and calendar months.
[a]Standardized rate estimates were calculated per 100,000 family-months assuming other predictors were at their sample-wide means.
[b]For non-biological children, age at birth of oldest child was calculated based on the first month of the child's appearance in the Active Duty Family data file.
[c]Race and ethnicity were drawn from the Active Duty Military Personnel Master data files. Race and ethnicity categories correspond to categories in the Active Duty Military Personnel Master data file.
[d]Other includes "Multiple race" and "Unspecified." "Multiple race" included service members with documented membership in more than one race category.
**p* < 0.05, ***p* < 0.01, ****p* < 0.001.

© 2026 Cozza SJ et al. *JAMA Network Open*.

eTable 4. Variance Inflation Factors for Covariates Included in Multivariable Logistic Regressions Predicting Child Maltreatment Types

	Neglect	Physical Abuse	Emotional Abuse	Sexual Abuse
	VIF[a]	VIF	VIF	VIF
**I. Sponsor socio-demographic characteristics**				
Sex				
Male	Ref	Ref	Ref	Ref
Female	1.12	1.12	1.12	1.12
Age at birth of oldest child (in years)[b]				
< 21	2.63	2.63	2.63	2.63
21 − 24	1.74	1.74	1.74	1.74
≥ 25	Ref	Ref	Ref	Ref
Current age (in years)				
< 21	1.39	1.39	1.39	1.39
21 − 24	2.17	2.17	2.17	2.17
25 − 29	1.83	1.83	1.83	1.83
30 − 34	Ref	Ref	Ref	Ref
35 − 39	1.77	1.77	1.77	1.77
≥ 40	2.52	2.52	2.52	2.52
Race[c]				
Asian	1.03	1.03	1.03	1.03
Black	1.14	1.14	1.14	1.14
Native American	1.02	1.02	1.02	1.02
Pacific Islander	1.01	1.01	1.01	1.01
White	Ref	Ref	Ref	Ref
Other[d]	1.17	1.17	1.17	1.17
Ethnicity[c]				
Non-Hispanic	Ref	Ref	Ref	Ref
Hispanic	1.19	1.19	1.19	1.19

© 2026 Cozza SJ et al. *JAMA Network Open*.

Education level				
Less than High School	1.07	1.07	1.07	1.07
High School or equivalent	4.23	4.23	4.23	4.23
Some college	2.63	2.63	2.63	2.63
College degree or higher	Ref	Ref	Ref	Ref
**II. Family characteristics**				
Marital status				
Military-civilian marriage	Ref	Ref	Ref	Ref
Dual-military marriage	1.05	1.05	1.05	1.05
Divorced/Separated/Widowed	1.08	1.08	1.08	1.08
Never married	1.15	1.15	1.15	1.15
Children at entry into active duty				
No	Ref	Ref	Ref	Ref
Yes	1.60	1.60	1.60	1.60
Number of dependent children				
1	1.54	1.54	1.54	1.54
2	Ref	Ref	Ref	Ref
3 or more	1.43	1.43	1.43	1.43
Age of youngest child				
0 − 1	5.39	5.38	5.38	Ref
2 − 4	4.57	4.57	4.57	1.45
5 − 12	3.94	3.94	3.94	2.03
≥ 13	Ref	Ref	Ref	1.76
**III. Military-related characteristics**				
Military service branch				
Navy	Ref	Ref	Ref	Ref
Army	1.88	1.88	1.88	1.88
Air Force	1.79	1.79	1.79	1.79
Marine Corps	1.43	1.43	1.43	1.43

© 2026 Cozza SJ et al. *JAMA Network Open*.

Military rank				
E1 to E3	1.43	1.43	1.43	1.43
E4 to E6	Ref	Ref	Ref	Ref
E7 to E9	1.65	1.65	1.65	1.65
Warrant officer	1.17	1.17	1.17	1.17
O1 to O3	1.93	1.93	1.93	1.93
O4 and higher	2.84	2.84	2.84	2.84
Deployment status				
Currently deployed	Ref	Ref	Ref	Ref
Ever deployed	1.86	1.86	1.86	1.86
Never deployed	1.95	1.95	1.95	1.95

Abbreviations: VIF, Variance Inflation Factor.

[a]VIFs were derived from linear regression models using identical coding and reference category specifications as the multivariable logistic models.

[b]For non-biological children, age at birth of oldest child was calculated based on the first month of the child's appearance in the Active Duty Family data file.

[c]Race and ethnicity were drawn from the Active Duty Military Personnel Master data files. Race and ethnicity categories correspond to categories in the Active Duty Military Personnel Master data file.

[d]Other includes “Multiple race” and “Unspecified.” “Multiple race” included service members with documented membership in more than one race category.

© 2026 Cozza SJ et al. *JAMA Network Open*.
