# Supplementary material for: Factors Associated With First Occurrences of Child Maltreatment in Military Families
Source: JAMA Netw Open. 2026 May 13;9(5):e2612199. doi: 10.1001/jamanetworkopen.2026.12199 (PMC13173382; doi:10.1001/jamanetworkopen.2026.12199)
Supplement: Supplement 2. — Data Sharing Statement [file jamanetwopen-e2612199-s002.pdf]

Data Sharing Statement

Cozza. Factors Associated With First Occurrences of Child Maltreatment in Military Families. *JAMA Netw Open*. Published May 13, 2026. doi:10.1001/jamanetworkopen.2026.12199

Data
Data available: No

Additional Information
Explanation for why data not available: We are unable to share data from the U.S. Military Family Advocacy Program's Central Registry describing child maltreatment incidents.
